# Efficacy and safety of atezolizumab plus bevacizumab in patients with portal hypertension for unresectable hepatocellular carcinoma

**DOI:** 10.1002/cam4.7025

**Published:** 2024-03-13

**Authors:** Takahiro Kinami, Shinsuke Uchikawa, Tomokazu Kawaoka, Shintaro Yamasaki, Masanari Kosaka, Yusuke Johira, Shigeki Yano, Kei Amioka, Kensuke Naruto, Kenji Yamaoka, Yasutoshi Fujii, Hatsue Fujino, Takashi Nakahara, Atsushi Ono, Eisuke Murakami, Wataru Okamoto, Masami Yamauchi, Daiki Miki, Masataka Tsuge, Shiro Oka

**Affiliations:** ^1^ Department of Gastroenterology, Graduate School of Biomedical and Health Sciences Hiroshima University Hiroshima Japan; ^2^ Department of Clinical Oncology Hiroshima University Hospital Hiroshima Japan

**Keywords:** adverse events, atezolizumab plus bevacizumab, esophagogastric variceal rupture, hepatocellular carcinoma, portal hypertension

## Abstract

**Aim:**

Atezolizumab plus bevacizumab combination therapy (Atezo + Beva) is used as the first‐line therapy for unresectable hepatocellular carcinoma (u‐HCC). Serious adverse events (AEs), including rupture of esophagogastric varices, have been seen during treatment. Therefore, the relationships of efficacy, safety, and portal hypertension (PH) were analyzed.

**Methods:**

A total of 146 patients with u‐HCC and Child‐Pugh Scores of 5–7 received Atezo + Beva. Prophylactic treatment for varices was performed for patients with the risk of rupture of varices before the start of Atezo + Beva. A propensity score‐matched cohort was created to minimize the risk of potential confounders. Efficacy was assessed in 41 propensity score‐matched pairs. AEs were assessed between patients without PH (*n* = 80) and with PH (*n* = 66).

**Results:**

In patients without PH and with PH, median overall survival was 18.4 months and 18.8 months (*p* = 0.71), and median progression‐free survival was 8.6 months and 5.8 months (*p* = 0.92), respectively. On the best radiological response evaluation for Response Evaluation Criteria in Solid Tumors, the objective response rate was 31.7% and 26.8% (*p* = 0.81), respectively. Variceal rupture occurred in three patients with PH, but there were no significant differences in the occurrence of variceal rupture (*p* = 0.090) and Grade 3–4 AEs between patients without and with PH.

**Conclusions:**

No significant differences in efficacy and safety were observed with PH. Prophylactic treatment for varices before the start of Atezo + Beva would allow treatment to continue relatively safely.

## 1 INTRODUCTION

Hepatocellular carcinoma (HCC) is the most frequently occurring primary liver malignancy and was reported to be a major cause of cancer death worldwide in 2020.^1^ HCC occurs in patients with chronic hepatitis or cirrhosis caused by hepatitis B virus (HBV), hepatitis C virus (HCV), excessive alcohol consumption, or diabetes mellitus.^2^ The prognosis of patients with unresectable hepatocellular carcinoma (u‐HCC) is poor.^3^, ^4^ Recently, systemic therapy for u‐HCC has achieved remarkable progress; sorafenib was approved as the first molecular targeted agent (MTA) in 2009,^5^ and lenvatinib was approved as a first‐line MTA in Japan in 2018.^6^ Additionally, regorafenib, ramucirumab, and cabozantinib were approved as second‐line MTAs.^7^, ^8^, ^9^ Atezolizumab plus bevacizumab combination therapy (Atezo + Beva) was approved as the first immune combination therapy in 2020.^10^ Because it was confirmed that Atezo + Beva maintained patients' quality of life and prolonged survival better than sorafenib in the IMbrave150 trial,^11^ Atezo + Beva was used as a first‐line treatment.

Bevacizumab inhibits tumor tissue angiogenesis and tumor growth by blocking the vascular endothelial growth factor (VEGF)‐mediated signaling pathway.^12^, ^13^

Some recent studies reported the efficacy and safety of Atezo + Beva for u‐HCC in Japanese real‐world clinical practice.^14^, ^15^, ^16^, ^17^ However, a certain number of patients experienced adverse events (AEs), such as hypertension, fatigue, and proteinuria, while receiving Atezo + Beva. Bleeding is a known risk in cancer patients receiving VEGF or VEGF receptor‐targeted therapies such as bevacizumab.^6^, ^18^ There were some cases of ruptured esophagogastric varices in the IMbrave150 trial.^11^ Patients with liver cirrhosis (LC) have a risk of upper gastrointestinal (UGI) bleeding of variceal and nonvariceal origin,^19^, ^20^ especially rupture of esophagogastric varices, which accounts for 70% of UGI bleeding events in patients with portal hypertension (PH).^21^, ^22^ PH is secondary to increased intrahepatic vascular resistance, opening of portal collateral vessels, and formation of VEGF‐associated neovessels.^23^, ^24^, ^25^ PH is a major complication of LC, causing esophagogastric varices, ascites, and hepatorenal syndrome, and it contributes significantly to prognosis. The worsening of esophagogastric varices should be noted when bevacizumab is used for HCC, because HCC and LC often coexist in the same patient.

Therefore, efficacy and safety (particularly UGI bleeding) of Atezo + Beva were compared between patients without PH and with PH using propensity score matching.

## 2 METHODS

### 2.1 Patients

A total of 172 patients agreed to participate in the study and were treated with Atezo + Beva for u‐HCC at Hiroshima University Hospital between October 2020 and February 2023. The inclusion criteria for this study were as follows: Child‐Pugh score 5–7 and Eastern Cooperative Oncology Group performance status (ECOG PS) 0 or 1.

Twenty‐two patients were excluded because their Child‐Pugh scores were >8, and six patients were excluded because of ECOG PS 2 or 3; thus, a total of 26 patients were excluded. Finally, a total of 146 patients met the criteria. All patients who participated in the original clinical trial provided written informed consent. The study protocol, any amendments to the protocol, and the informed consent form were reviewed and approved by the relevant Institutional Review Board/Independent Ethics Committee. The study was conducted in accordance with the principles of the Declaration of Helsinki and Good Clinical Practice Guidelines.

Patients positive for HBV surface antigens were considered to have HBV‐induced HCC, and those positive for anti‐HCV antibodies were considered to have HCV‐induced HCC. Patients who were negative for HBV surface antigen and negative for HCV antibody were considered to have hepatocellular carcinoma due to non‐viral hepatitis.

Hepatic reserve was assessed by the Child‐Pugh score and the modified albumin–bilirubin (mALBI) grade.

### 2.2 Definition of portal hypertension

PH was defined as having any of esophagogastric varices, splenomegaly, or portosystemic collateral vessels at the start of Atezo + Beva. All patients were evaluated for esophagogastric varices by esophagogastroduodenoscopy (EGD) prior to Atezo + Beva treatment.

### 2.3 Definition of esophagogastric varices

**FIGURE 1:**
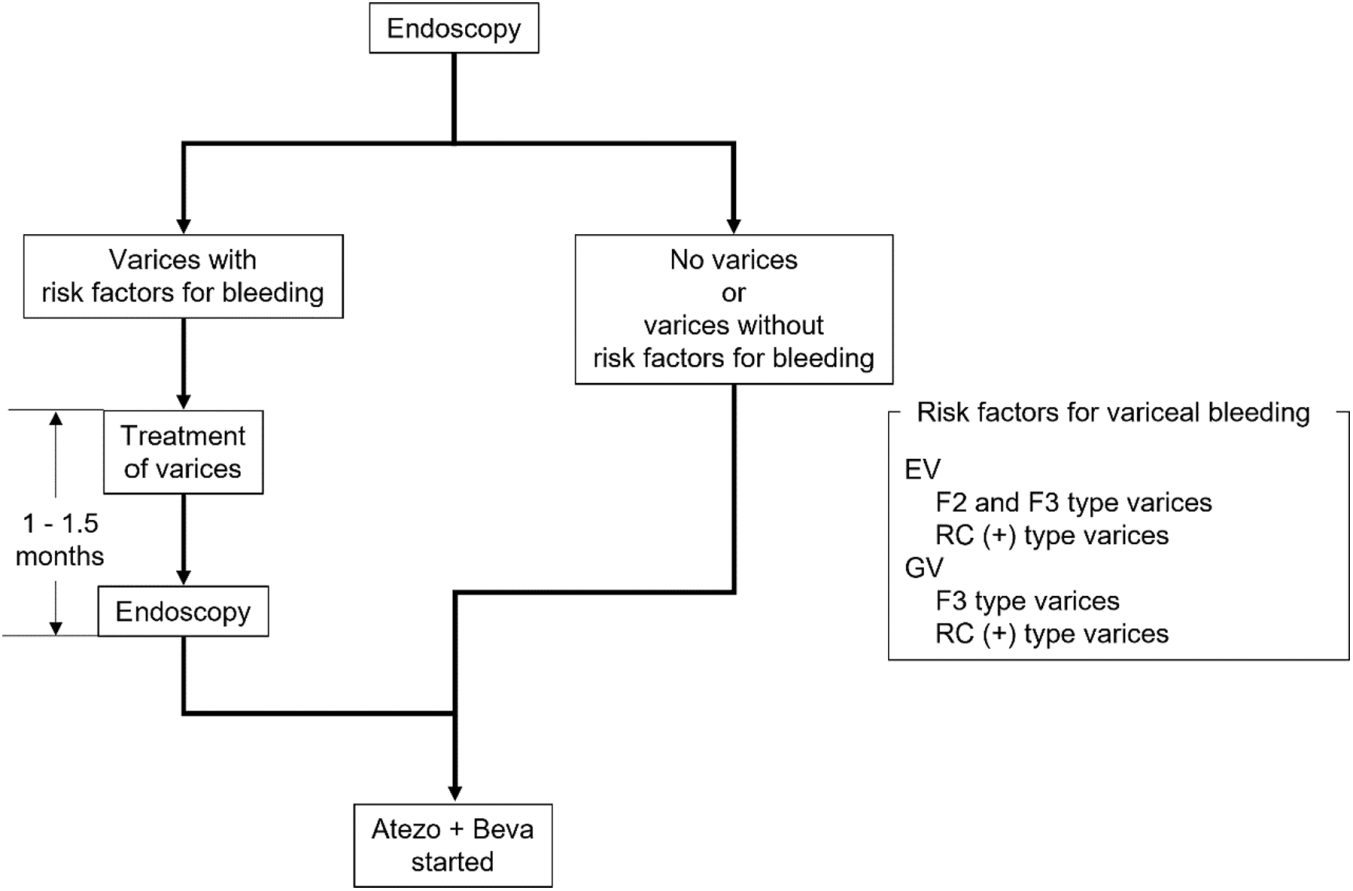
Flow chart of endoscopic evaluation of esophageal varices (EV) and gastric varices (GV) before the start of atezolizumab plus bevacizumab combination therapy (Atezo + Beva). F, form; RC, red color sign.

The endoscopic findings of varices were evaluated according to the classification system of the Japanese Society for Portal Hypertension and Esophageal Varices.^26^ The form (F) of varices was classified as follows: complete eradication after treatment (F0), small straight (F1), enlarged tortuous (F2), and large coil‐shaped (F3). The red color sign (RC) was also classified according to the criteria of the Japanese Society for Portal Hypertension and Esophageal Varices.^26^ Esophagogastric varices greater than F0 were considered varices. As a rule in this study, in cases with the following risk factors for variceal bleeding (cases with esophageal varices (EV): F2, F3, or RC(+), cases with gastric varices (GV): F3 or RC(+)), the varices were to be treated by endoscopic variceal ligation (EVL), endoscopic injection sclerotherapy (EIS), or balloon‐occluded retrograde transvenous obliteration (B‐RTO), and 1–1.5 months later, EGD was performed to confirm the reduction of varices before the start of Atezo + Beva treatment (Figure 1).

### 2.4 Definition of splenomegaly

**FIGURE 2:**
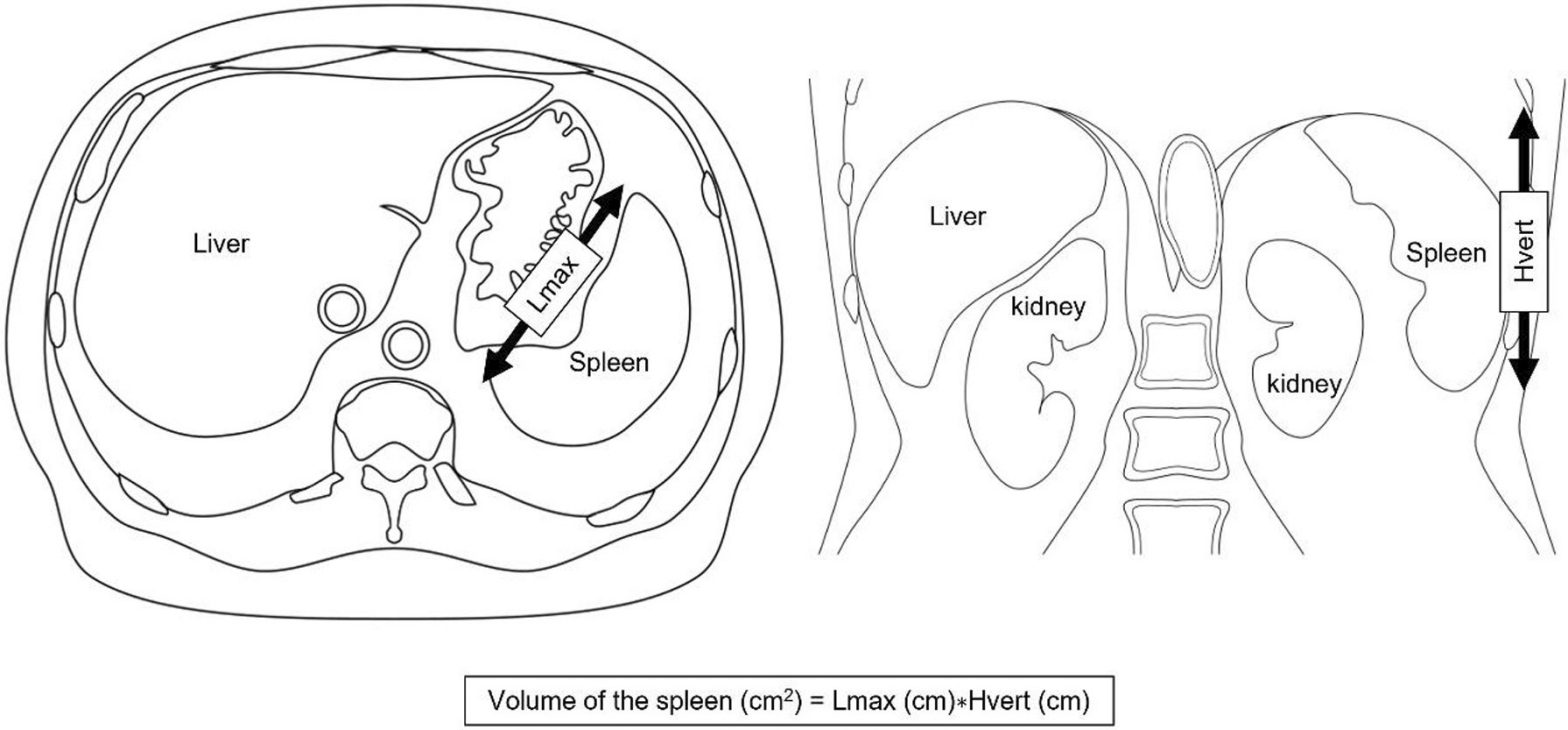
The method for measuring the volume of the spleen using maximal length (Lmax) in the axial image and vertical height (Hvert) in the coronal image.

The coefficient calculated from maximal length, vertical height, and hilar thickness correlates with spleen volume and can be used to monitor splenic volume. The most suitable for quick splenomegaly screening is the two‐dimensional coefficient (maximal length × vertical height), with the cutoff of 115 cm^2^.^27^ Splenomegaly was defined as spleen volume >115 cm^2^ (Figure 2).

### 2.5 Definition of portosystemic collateral vessels

The left gastric vein, posterior gastric vein, short gastric vein, paraesophageal vein, paraumbilical vein, gastrorenal shunt, and splenorenal shunt were associated with portal hemodynamics. These vessels were assessed by dynamic computed tomography (CT). The maximum diameter of these vessels was measured in all cases except those without dynamic CT at the start of Atezo + Beva. Based on previous reports, the cutoff diameters of the portosystemic collateral vessels, the left gastric vein, posterior gastric vein, short gastric vein, paraesophageal vein, paraumbilical vein, gastrorenal shunt, and splenorenal shunt, were defined as 6, 4, 2, 4, 3, 13, and 13 mm, respectively.^28^ Portosystemic collateral vessels were defined as having any vessels exceeding the cutoff value.

### 2.6 Treatments

Atezo + Beva, consisting of atezolizumab 1200 mg intravenously plus bevacizumab 15 mg/kg, was administered every 3 weeks. The Common Terminology Criteria for Adverse Events version 5.0 were used to evaluate AEs. Bevacizumab was interrupted if AEs such as proteinuria or hypertension occurred. Atezo + Beva was discontinued in the event of progressive disease following treatment, decrease in ECOG PS or hepatic reserve, or unacceptable or severe treatment‐related AEs.

### 2.7 Assessment of treatment response

Radiological response assessment was performed by dynamic CT/MRI every 1.5 months. Treatment response was evaluated by the Response Evaluation Criteria in Solid Tumors (RECIST) version 1.1 and modified Response Evaluation Criteria in Solid Tumors (mRECIST) guidelines. The overall response rate (ORR) and disease control rate (DCR) were evaluated based on these guidelines.

### 2.8 Follow‐up of esophagogastric varices by EGD

We believed that EGD should be performed regularly, and suggested patients have follow‐up EGD within 6 months to a year after start of Atezo + Beva. We strongly suggested follow‐up EGD if dynamic CT showed worsening of esophagogastric varices or if findings in EGD at the time of start of Atezo + Beva indicated a high risk of worsening of esophagogastric varices.

### 2.9 Statistical analysis

We conducted a propensity score‐matched cohort was calculated to minimize the risk of potential confounders, considering the differences in efficacy and safety‐related characteristics between patients without PH and with PH. The propensity score for each patient was estimated by a logistic regression model. The matching variables included serum albumin, serum total bilirubin, prothrombin activity, and extrahepatic metastasis (EHM). For matching, 1:1 nearest neighbor matching was used, with a caliper width of 0.2 standard deviations.

Fisher's exact test or the chi‐squared test, the Mann–Whitney *U*‐test, and the Kaplan–Meier method were used for statistical analysis.

A *p*‐value less than 0.05 was considered to indicate a significant difference. All statistical analyses were carried out using Predictive Analytics Software R version 4.1.2.

## 3 RESULTS

### 3.1 Clinical characteristics of patients before and after propensity score matching

**FIGURE 3:**
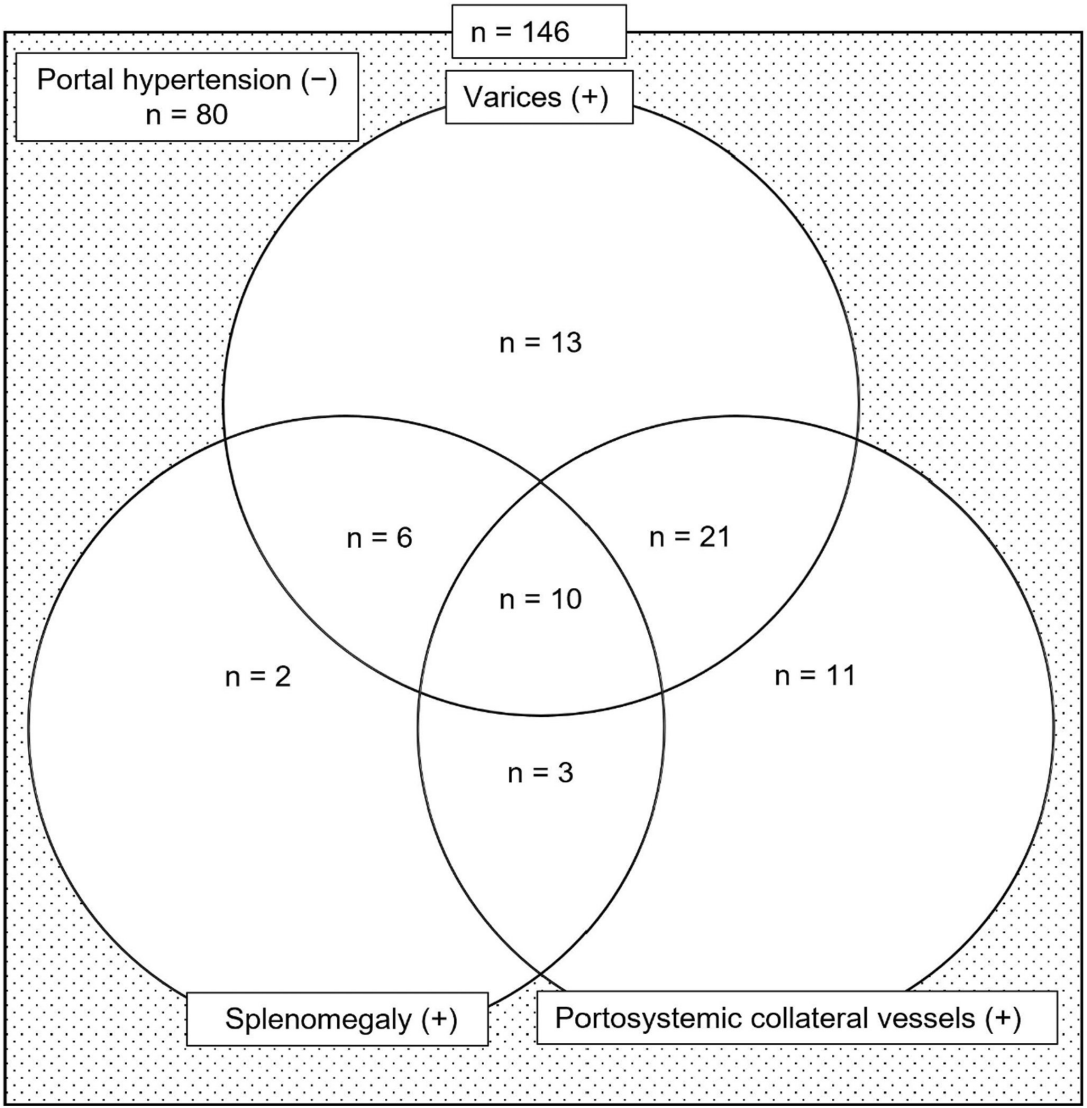
Diagram of the numbers of patients with varices, splenomegaly, and portosystemic collateral vessels at the start of atezolizumab plus bevacizumab combination therapy.

The numbers of patients without PH (PH(−)) and with PH (PH(+)) were 80 and 66, respectively, and the following is a diagram of each factor for patients with PH (Figure 3). Of the PH(+) patients, 50 had varices, 21 had splenomegaly, and 45 had portosystemic collateral vessels.

The patients' background characteristics are shown in Table 1.

**TABLE 1:** Comparison of clinical characteristics at the start of atezolizumab plus bevacizumab combination therapy between patients without portal hypertension (PH(−)) and with portal hypertension (PH(+)) at the time of study entry before and after propensity score matching.

Characteristic	Before matching median (quartiles) or patients, *n*			After matching median (quartiles) or patients, *n*		
	PH(−) (*n* = 80)	PH(+) (*n* = 66)	*p* Value*	PH(−) (*n* = 41)	PH(+) (*n* = 41)	*p* Value*
Age (years)^a^	74 (68–79)	71 (67–79)	0.55	73 (68–82)	72 (66–82)	0.74
Sex (male/female), *n*	66/14	51/15	0.53	35/6	32/9	0.57
Etiology (HBV/HCV/non‐viral), *n*	12/22/46	8/30/28	0.081	7/13/21	5/23/13	0.090
Child‐Pugh score (5/6/7), *n*	49/25/6	28/24/14	0.021	25/14/2	23/13/5	0.62
ECOG PS (0/1), *n*	74/6	62/4	1	38/3	38/3	1
Line of Atezo + Beva (1st/2nd/3rd/4th/5th), *n*	48/27/0/4/1	49/11/2/3/1	0.063	26/14/0/1/0	28/8/1/3/1	0.27
Previous treatment prior to Atezo + Beva (absent/present), *n*	18/62	10/56	0.30	9/32	4/37	0.23
Resection (absent/present), *n*	41/39	35/31	0.87	22/19	21/20	1
Ablation (absent/present), *n*	78/2	59/7	0.080	39/2	34/7	0.16
Chemoembolization (absent/present), *n*	35/45	20/46	0.12	17/24	11/30	0.24
Stereotactic body radiation therapy (absent/present), *n*	79/1	61/5	0.091	40/1	38/3	0.62
Observation period (month)^a^	12.8 (8.2–18.6)	11.0 (6.7–17.6)	0.28	13.7 (5.7–16.9)	11.8 (6.9–18.8)	0.71
ALBI score^a^	−2.57 (−2.75 to −2.19)	−2.31 (−2.56 to −1.92)	<0.001	−2.52 (−2.69 to −2.13)	−2.48 (−2.69 to −2.14)	0.97
Modified ALBI grade (1/2a/2b/3), *n*	37/20/23/0	16/20/28/2	0.016	16/11/14/0	15/13/13/0	0.92
Serum albumin (g/dL)^a^	3.9 (3.4–4.1)	3.6 (3.3–3.9)	0.019	3.8 (3.3–4.0)	3.8 (3.4–4.1)	0.96
Serum total bilirubin (mg/dL)^a^	0.7 (0.5–0.9)	0.9 (0.7–1.1)	<0.001	0.7 (0.6–1.1)	0.8 (0.7–0.9)	1
Prothrombin activity (%)^a^	97 (88–103)	84 (76–94)	<0.001	91 (86–100)	92 (82–97)	0.57
Ascites (none/mild/moderate to severe), *n*	74/6/0	60/5/1	0.75	40/1/0	38/2/1	0.62
Encephalopathy (none/Grade I–II/Grade III–IV), *n*	80/0/0	66/0/0	1	41/0/0	41/0/0	1
Serum ammonia level (μg/dL)^a^	26 (17–34)	33 (23–51)	<0.001	26 (18–35)	30 (21–41)	0.30
Size of main hepatic tumor (mm)^a^	37 (16–63)	30 (17–49)	0.42	40 (22–70)	30 (15–45)	0.25
Relative tumor size (<50%/>50%), *n*	76/4	60/6	0.35	38/3	36/5	0.71
MVI (absent/present), *n*	68/12	47/19	0.067	33/8	28/13	0.31
Vp (1/2/3/4), *n*	0/3/6/2	3/4/4/5	0.31	0/2/5/0	3/1/4/3	0.20
Vv (1/2/3), *n*	0/1/3	2/1/3	0.71	0/1/2	2/1/3	1
EHM (absent/present), *n*	49/31	53/13	0.018	30/11	30/11	1
BCLC stage (A/B/C), *n*	4/39/37	5/33/28	0.81	3/21/17	4/16/21	0.64
Serum AFP level (ng/mL)^a^	24.2 (4.3–351)	33 (4.3–176)	0.93	21.8 (4.9–216)	38.5 (4.5–240)	0.53
Serum DCP level (mAU/mL)^a^	362 (55–4893)	358 (110–1918)	0.98	418 (112–3039)	567 (109–2705)	0.30
Esophageal and/or gastric varices (absent/present), *n*	80/0	16/50	<0.001	41/0	10/31	<0.001
Form of esophageal varices (absent/F0/F1/F2/F3), *n*	80/0/0/0/0	19/2/37/8/0	<0.001	41/0/0/0/0	13/2/21/5/0	<0.001
Red color sign of esophageal varices (RC0/RC1/RC2), *n*	80/0/0	57/7/2	<0.001	41/0/0	37/3/1	0.12
Form of gastric varices (absent/F0/F1/F2/F3), *n*	80/0/0/0/0	51/0/8/6/1	<0.001	41/0/0/0/0	31/0/5/5/0	0.001
Red color sign of gastric varices (RC0/RC1), *n*	80/0	64/2	0.20	41/0	41/0	1
Splenomegaly (absent/present), *n*	80/0	45/21	<0.001	41/0	28/13	<0.001
Volume of the spleen (cm^2^)^a^	59 (45–78)	97 (71–128)	<0.001	65 (48–79)	93 (71–131)	<0.001
Portosystemic collateral vessels (absent/present), *n*	80/0	21/45	<0.001	41/0	14/27	<0.001
Diameter of left gastric vein (mm)^a^	2 (1–3)	3 (1–4)	0.014	1 (1–2)	3 (1–4)	<0.001
Diameter of posterior gastric vein (mm)^a^	1 (1–2)	2 (1–4)	<0.001	1 (1–2)	1 (1–3)	0.039
Diameter of short gastric vein (mm)^a^	1 (1–1)	1 (1–1)	0.0034	1 (1–1)	1 (1–1)	0.047
Diameter of paraesophageal vein (mm)^a^	1 (1–1)	2 (1–4)	<0.001	1 (1–1)	2 (1–4)	<0.001
Diameter of paraumbilical vein (mm)^a^	0 (0–0)	0 (0–0)	0.0034	0 (0–0)	0 (0–0)	0.093
Diameter of gastrorenal shunt (mm)^a^	0 (0–0)	0 (0–0)	0.29	0 (0–0)	0 (0–0)	0.33
Diameter of splenorenal shunt (mm)^a^	0 (0–0)	0 (0–0)	<0.001	0 (0–0)	0 (0–0)	0.012

Abbreviations: AFP, alpha‐fetoprotein; ALBI, albumin–bilirubin; Atezo + Beva, atezolizumab plus bevacizumab combination therapy; BCLC, Barcelona clinic liver cancer; DCP, des‐γ‐carboxy prothrombin.; ECOG PS, Eastern Cooperative Oncology Group performance status; EHM, extrahepatic metastasis; HBV, hepatitis B virus infection; HCV, hepatitis C virus infection; MVI, microvascular invasion.

^a^ Median (quartiles).

* Fisher's exact test or chi‐squared test, Mann–Whitney *U*‐test.

In PH(−) patients, the median age was 74 years, and the observation period was 12.8 months. In PH(+) patients, the median age was 71 years, and the observation period was 11.0 months. There were no significant differences in age, sex, etiology, ECOG PS, line of Atezo + Beva, previous treatment prior to Atezo + Beva (resection, ablation, chemoembolization, and stereotactic body radiation therapy), and observation period between PH(−) and PH(+) patients. However, the Child‐Pugh score, mALBI grade, serum albumin, serum total bilirubin, prothrombin activity, and serum ammonia levels were significantly worse in PH(+) patients than in PH(−) patients. In contrast, the number of EHM was significantly greater in PH(−) than in PH(+) patients. To match tumor factors and hepatic reserve, 41 matched pairs of patients with and without PH were created using propensity score matching. After matching, there were no significant differences in baseline characteristics between the two groups except for PH.

In both PH(−) and PH(+) patients, there were 30 patients without EHM and 11 patients with EHM, with no significant difference in EHM after matching. Similarly, there were no significant differences in Barcelona clinic liver cancer (BCLC) stage, microvascular invasion (MVI), and tumor markers after matching between PH(−) and PH(+) patients. Moreover, there were no significant differences in the levels of Vp and Vv.

In addition, the albumin–bilirubin index (ALBI) score was −2.52 (−2.69 to −2.13) and − 2.48 (−2.69 to −2.14), respectively, with no significant difference (*p* = 0.97) after matching. There were also no significant differences in prothrombin activity and serum ammonia levels, indicating that matching was able to align tumor factors and hepatic reserve.

### 3.2 Survival and treatment response

In PH(−) and PH(+) patients, the median number of atezolizumab cycles was 11 and 8, respectively, with no significant difference (*p* = 0.28). Similarly, the median number of bevacizumab cycles was 9 and 7, respectively, with no significant difference (*p* = 0.23).

**FIGURE 4:**
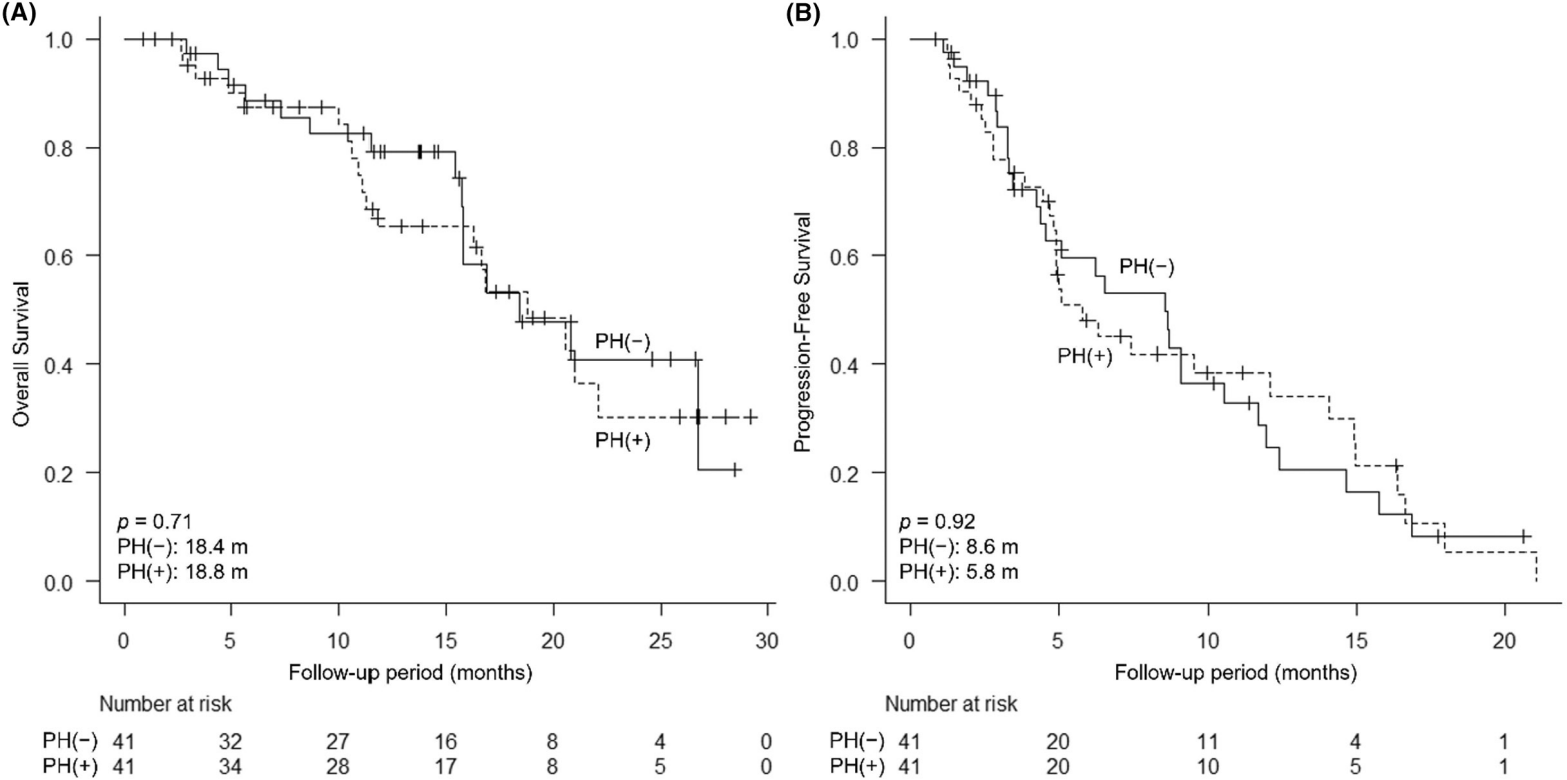
Comparison of overall survival (OS) and progression‐free survival (PFS) from the start of atezolizumab plus bevacizumab (Atezo + Beva) between patients without portal hypertension (PH(−)) and with portal hypertension (PH(+)) after propensity score matching. (A) OS from the start of Atezo + Beva (PH(−) 18.4 months, PH(+) 18.8 months, *p* = 0.71). (B) PFS from the start of Atezo + Beva (PH(−) 8.6 months, PH(+) 5.8 months, *p* = 0.92).

After propensity score matching, the median OS of the PH(−) and PH(+) patients was 18.4 months and 18.8 months, respectively (Figure 4), with no significant difference (*p* = 0.71). The median PFS of PH(−) and PH(+) was 8.6 months and 5.8 months, respectively, with no significant difference between PH(−) and PH(+) patients (*p* = 0.92). Before propensity score matching, the median OS of the PH(−) and PH(+) patients was 26.7 months and 18.8 months, respectively, with no significant difference (*p* = 0.11). The median PFS of PH(−) and PH(+) was 8.7 months and 8.2 months, respectively, with no significant difference (*p* = 0.69).

Table 2 shows the comparison of the best radiological response evaluation after propensity score matching between PH(−) and PH(+) patients by RECIST and mRECIST. On the best radiological response evaluation by RECIST of PH(−) and PH(+) patients, 2 patients (4.9%) and 1 patient (2.4%) had complete response (CR), 11 patients (26.8%) and 10 patients (24.4%) had partial response (PR), 15 patients (36.6%) and 20 patients (48.8%) had stable disease (SD), and 9 patients (22.0%) and 10 patients (24.4%) had progressive disease (PD), respectively. There were no significant differences in ORR and DCR (ORR 31.7% and 26.8% (*p* = 0.81), DCR 68.3% and 75.6% (*p* = 0.62), respectively). On mRECIST, there were also no significant differences in ORR and DCR (ORR 48.8% and 46.3% (*p* = 1), DCR 68.3% and 75.6% (*p* = 0.62), respectively). Before propensity score matching, there were no significant differences on RECIST in ORR and DCR (ORR 31.3% and 36.4% (*p* = 0.60), DCR 70.0% and 81.8% (*p* = 0.12), respectively). On mRECIST, there were also no significant differences in ORR and DCR (ORR 43.8% and 50.0% (*p* = 0.51), DCR 71.3% and 81.8% (*p* = 0.17), respectively).

**TABLE 2:** Comparison of radiological best responses to atezolizumab plus bevacizumab combination therapy after propensity score matching between patients without portal hypertension (PH(−)) and with portal hypertension (PH(+)) by Response Evaluation Criteria in Solid Tumors (RECIST) and modified RECIST (mRECIST).

% (*n*)	RECIST			mRECIST		
	PH(−) (*n* = 41)	PH(+) (*n* = 41)	*p* Value *	PH(−) (*n* = 41)	PH(+) (*n* = 41)	*p* Value *
CR	4.9 (2)	2.4 (1)		7.3 (3)	7.3 (3)	
PR	26.8 (11)	24.4 (10)		41.5 (17)	39.0 (16)	
SD	36.6 (15)	48.8 (20)		19.5 (8)	29.3 (12)	
PD	22.0 (9)	24.4 (10)		22.0 (9)	22.0 (9)	
NE	9.8 (4)	0(0)		9.8 (4)	2.4 (1)	
ORR	31.7 (13)	26.8 (11)	0.81	48.8 (20)	46.3 (19)	1
DCR	68.3 (28)	75.6 (31)	0.62	68.3 (28)	75.6 (31)	0.62

Abbreviations: CR, complete response; DCR, disease control rate; NE, not evaluated; ORR, overall response rate; PD, progressive disease; PR, partial response; SD, stable disease.

* Fisher's exact test or chi‐squared test.

### 3.3 Adverse events

AEs that occurred during treatment are listed in Table 3. In PH(−) and PH(+) patients, the most common any grade AEs were hypertension (65 cases [81.3%] and 56 cases [84.8%], respectively), followed by fatigue (62 cases [77.5%] and 49 cases [74.2%], respectively), proteinuria (55 cases [68.8%] and 37 cases [56.1%], respectively), pruritus (45 cases [56.3%] and 42 cases [63.6%], respectively), and decreased appetite (45 cases [56.3%] and 39 cases [59.1%], respectively). There were no significant differences in the incidences of these AEs between PH(−) and PH(+) patients. In contrast, the incidences of ascites, portal vein thrombus, and pyrexia were significantly higher in PH(+) patients. Regarding Grade 3 or 4 AEs in PH(−) and PH(+) patients, the most common AEs were hypertension (22 cases [27.5%] and 13 cases [19.7%], respectively), followed by proteinuria (14 cases [17.5%] and 10 cases [15.2%], respectively), and increased aspartate aminotransferase (AST) or alanine aminotransferase (ALT) (5 cases [6.3%] and 3 cases [4.5%], respectively), and there were no significant differences in the incidence of these AEs between PH(−) and PH(+) patients. No other AEs were significantly different between PH(−) and PH(+) patients. A few cases of variceal rupture and gastrointestinal bleeding (including stomach ulcer and colonic diverticular bleeding) that appeared to be bevacizumab‐related AEs were observed, but there were no significant differences between PH(−) and PH(+) patients. Hepatic encephalopathy was not observed in PH(−) patients. However, 1 case of Grade 2 and 2 cases of Grade 3 assessed by West Haven Criteria for hepatic encephalopathy were observed in PH(+) patients, but there was no significant difference in the incidence of hepatic encephalopathy between PH(−) and PH(+) patients.

**TABLE 3:** Comparison of adverse events associated with atezolizumab plus bevacizumab combination therapy between patients without portal hypertension (PH(−)) and with portal hypertension (PH(+)).

Event % (*n*)	Any grade			Grade 3 or 4		
	PH(−) (*n* = 80)	PH(+) (*n* = 66)	*p* Value*	PH(−) (*n* = 80)	PH(+) (*n* = 66)	*p* Value*
Varix rupture	0 (0)	4.5 (3)	0.090	0 (0)	4.5 (3)	0.090
Stomach ulcer	1.3 (1)	6.1 (4)	0.18	1.3 (1)	1.5 (1)	1
Gastric antral vascular ectasia	0 (0)	1.5 (1)	0.45	0 (0)	0 (0)	1
Colonic diverticular bleeding	1.3 (1)	1.5 (1)	1	0 (0)	1.5 (1)	0.45
Gastrointestinal perforation	1.3 (1)	0 (0)	1	1.3 (1)	0 (0)	1
Ascites	11.3 (9)	28.8 (19)	0.011	1.3 (1)	3.0 (2)	0.59
Portal vein thrombus	1.3 (1)	9.1 (6)	0.046	1.3 (1)	3.0 (2)	0.59
Stomatitis	1.3 (1)	3.0 (2)	0.59	0 (0)	1.5 (1)	0.45
Interstitial pneumonia	3.8 (3)	1.5 (1)	0.63	0 (0)	0 (0)	1
Fatigue	77.5 (62)	74.2 (49)	0.70	3.8 (3)	4.5 (3)	1
Decreased appetite	56.3 (45)	59.1 (39)	0.74	0 (0)	4.5 (3)	0.090
Rash	10.0 (8)	6.1 (4)	0.26	2.5 (2)	1.5 (1)	1
Pruritus	56.3 (45)	63.6 (42)	0.40	5.0 (4)	4.5 (3)	1
Hypertension	81.3 (65)	84.8 (56)	0.66	27.5 (22)	19.7 (13)	0.33
Proteinuria	68.8 (55)	56.1 (37)	0.12	17.5 (14)	15.2 (10)	0.82
Acute renal failure	10.0 (8)	13.6 (9)	0.61	1.3 (1)	0 (0)	1
Fever	10.0 (8)	22.7 (15)	0.042	0 (0)	3.0 (2)	0.20
Edema	38.8 (31)	45.5 (30)	0.50	6.3 (5)	1.5 (1)	0.22
Diarrhea	23.8 (19)	22.7 (15)	1	1.3 (1)	1.5 (1)	1
Increased AST or ALT	13.8 (11)	21.2 (14)	0.27	6.3 (5)	4.5 (3)	0.73
Thrombocytopenia	2.5 (2)	10.6 (7)	0.079	1.3 (1)	4.5 (3)	0.33
Thyroid dysfunction	7.5 (6)	9.1 (6)	0.77	0 (0)	0 (0)	1
Adrenal insufficiency	3.8 (3)	3.0 (2)	1	0 (0)	0 (0)	1

Abbreviations: ALT, alanine aminotransferase; AST, aspartate aminotransferase.

* Fisher's exact test or chi‐squared test.

### 3.4 Interruption of Beva and discontinuation of Atezo + Beva due to adverse events

In PH(−) and PH(+) patients, Beva was interrupted (41 cases [51.3%] and 43 cases [65.2%], respectively) by any reasons. There were no significant differences between PH(−) and PH(+) patients both before and after matching, respectively (*p* = 0.097 and *p* = 0.11). Moreover, of the PH(−) patients who had interrupted Beva (*n =* 41), 29 cases [70.7%] had interrupted Beva due to AEs. Of the PH(+) patients who had interrupted Beva (*n =* 43), 27 cases [62.8%] had interrupted Beva due to AEs. There were also no significant differences between PH(−) and PH(+) patients both before and after matching, respectively (*p* = 0.49 and *p* = 0.24). Proteinuria was the most cause of interruption of Beva, accounting for 60.0% of PH(−) patients and 37.0% of PH(+) patients. There were also no significant differences in any events between PH(−) and PH(+) patients. Other reasons besides AEs included decreased hepatic reserve, bone fractures, tooth extractions, treatment of varices, and COVID‐19. The median cycles of Beva administered before the first interruption of Beva in PH(−) and PH(+) patients who had interrupted Beva due to AEs were 3.5 and 3.0, respectively. No statistically significant difference was observed between PH(−) and PH(+) patients (*p* = 0.37).

In addition, 64 cases [80.0%] discontinued Atezo + Beva in PH(−) patients. Among them, 8 patients were due to AEs, including 5 patients with increased AST or ALT, 1 patient with gastrointestinal perforation, 1 patient with acute renal failure, and 1 patient with interstitial pneumonia. Interstitial pneumonia was Grade 2, but Atezo + Beva was discontinued in consultation with the respiratory medicine department. Fifty‐one cases [77.3%] discontinued Atezo + Beva in PH(+) patients. Among them, 4 patients were due to AEs, including 3 patients with increased AST or ALT, 1 patient with stomatitis. Discontinuation of Atezo + Beva by AEs did not differ significantly between PH(−) and PH(+) patients (*p* = 0.54). Therefore, there were no significant differences in impact of PH on interruption of Beva and discontinuation due to AEs.

### 3.5 Progression of esophagogastric varices during Atezo + Beva

**FIGURE 5:**
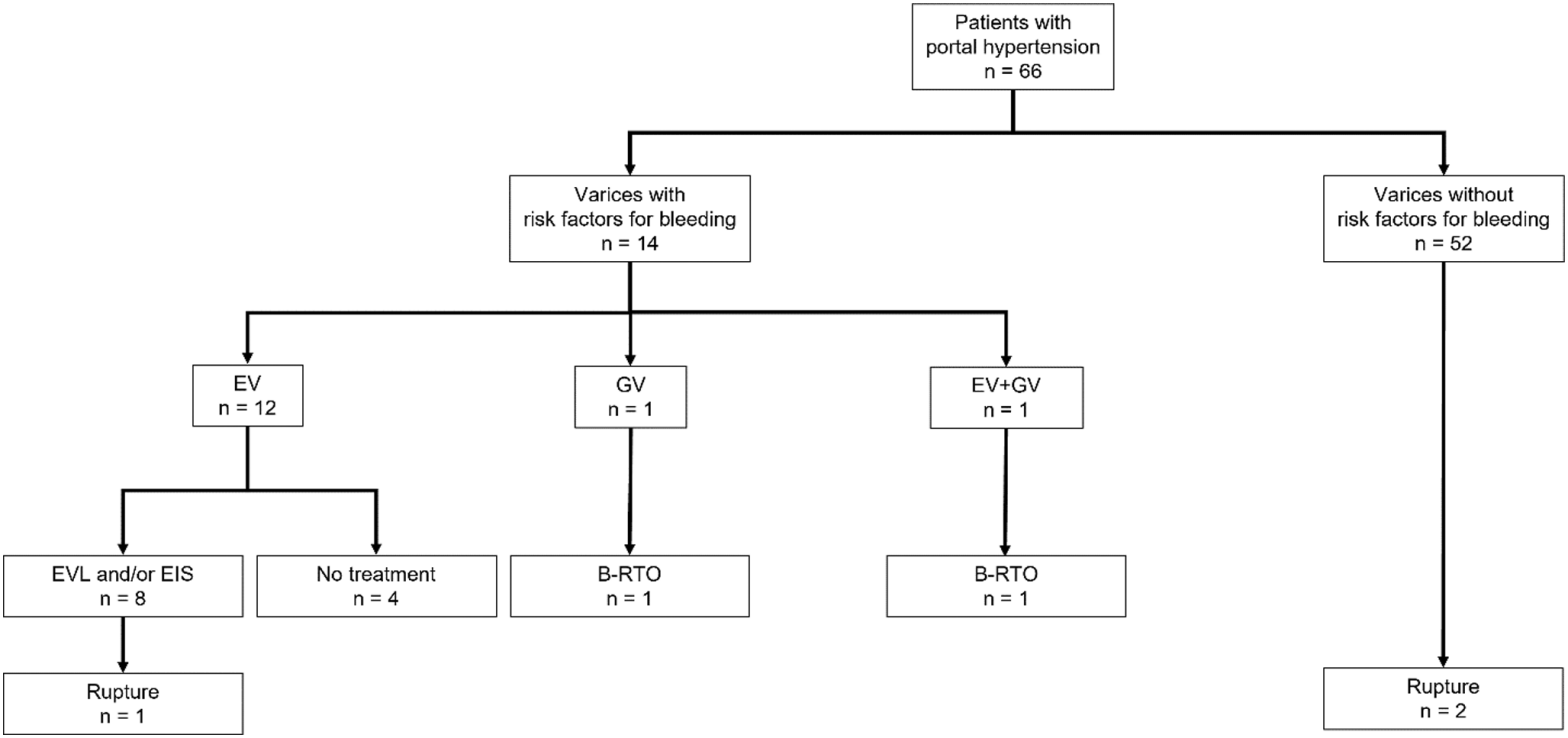
Flow chart of the numbers of patients with portal hypertension with varices, treatment of varices, and rupture. B‐RTO, balloon‐occluded retrograde transvenous obliteration; EIS, endoscopic injection sclerotherapy; EV, esophageal varices; EVL, endoscopic variceal ligation; GV, gastric varices.

Of the PH(+) patients (*n =* 66), 14 (12 had EV, 1 had GV, and 1 had EV + GV) had a risk of variceal rupture on EGD before the start of Atezo + Beva (Figure 5). Of the 12 patients with EV, 8 underwent EVL and/or EIS prior to the start of Atezo + Beva and showed variceal reduction; only 1 patient had variceal rupture during Atezo + Beva treatment. The other 4 patients with EV did not undergo treatment for varices prior to the start of Atezo + Beva because they prioritized treatment of HCC. One patient with GV underwent B‐RTO prior to the start of Atezo + Beva. One patient with EV + GV also underwent B‐RTO prior to the start of Atezo + Beva, but refused additional EVL or EIS.

We suggested patients have follow‐up EGD within 6 months to a year after start of Atezo + Beva, but some patients refused follow‐up EGD because of the invasion. Of the PH(−) patients (*n =* 80), 29 cases [36.3%] had underwent follow‐up EGD. Of these, 4 cases [13.8%] had worsening of esophagogastric varices with the bleeding risk. The median time to follow‐up EGD was 303 days. In contrast, of the PH(+) patients (*n =* 66), 39 cases [57.4%] had underwent follow‐up EGD. Of these, 22 cases [56.4%] had worsening of esophagogastric varices with the bleeding risk. The median time to follow‐up EGD was 173 days. Although these are reference findings because only some patients could be followed up, there were more worsening of esophagogastric varices significantly in PH(+) patients than in PH(−) patients in the follow‐up EGD (*p* < 0.001). In addition, of the 52 patients who did not have a risk of variceal rupture at the start of Atezo + Beva, two were found to have ruptured during treatment. In total, three cases of variceal rupture were observed. For these three cases of variceal rupture, their characteristics at baseline and those of the varices are summarized in Table 4.

**TABLE 4:** Characteristics of the three patients with ruptured varices.

Case	Age (years)	Sex	Etiology	Child‐Pugh class, score	Modified ALBI grade	Splenomegaly	Portosystemic collateral vessels	History of treatment for varices prior to HCC being noted	EV	GV	Treatment for varices for start of Atezo + Beva	Treatment for varices while receiving Atezo + Beva	Rupture site	Cycle of Atezo + Beva administered before varices ruptured
1	62	Male	HBV	B, 7	2b	+	−	EVL + EIS	F1RC1	−	EVL	EVL	EV	16
2	65	Male	Alcohol	A, 6	2a	+	+	EVL	F1RC0	F1RC0	−	−	GV	8
3	65	Male	HCV	A, 5	2a	+	+	EVL + EIS	F1RC0	−	−	−	EV	17

Abbreviations: ALBI, albumin–bilirubin index; Atezo + Beva, atezolizumab plus bevacizumab combination therapy; EIS, endoscopic injection sclerotherapy; EV, esophageal varices; EVL, endoscopic variceal ligation; F, form; GV, gastric varices; HBV, hepatitis B virus; HCC, hepatocellular carcinoma; HCV, hepatitis C virus; RC, red color sign.

## 4 DISCUSSION

Currently, Atezo + Beva is used as the first‐line immunotherapy for u‐HCC.

Bevacizumab inhibits tumor tissue angiogenesis and tumor growth by blocking the VEGF‐mediated signaling pathway.^12^, ^13^ Bevacizumab has been reported to be associated with proteinuria and hypertension due to nephrotoxicity and gastrointestinal bleeding.^29^, ^30^ With respect to serious gastrointestinal bleeding, 3 cases of variceal rupture (2.1%), 2 cases of bleeding from stomach ulcer (1.4%), and 1 case of colonic diverticular bleeding (0.7%) were seen in the present study. It has been reported that the incidence of UGI bleeding was 7% with Atezo + Beva in IMbrave 150.^11^ Two cases of rapid increase of EV after Atezo + Beva have been reported.^31^ Another study reported five cases of UGI bleeding and two cases of exacerbated varices during Atezo + Beva treatment.^32^ AEs of bleeding, such as ruptured varices, require temporary interruption of Atezo + Beva therapy, and it has been reported that, once gastrointestinal bleeding occurs, continuation of Atezo + Beva is difficult in some cases for the following reasons: rapid deterioration of hepatic reserve, rapid growth of HCC, and acquired resistance against Atezo + Beva.^33^, ^34^ Therefore, the present study focused on PH, which can be associated with varices, and efficacy and safety were compared between PH(−) and PH(+) patients.

There were no significant differences in clinical characteristics such as age, line of Atezo + Beva, and observation period between PH(−) and PH(+) patients. However, hepatic reserve was significantly worse in PH(+) patients than in PH(−) patients at the start of Atezo + Beva. In contrast, the number of EHM was significantly greater in PH(−) than in PH(+) patients. Better hepatic reserve, such as mALBI Grade 1 or 2a, is thought to indicate a better condition for obtaining a sufficient outcome with Atezo + Beva for u‐HCC patients.^35^ Therefore, tumor factors and hepatic reserve were matched in both groups using propensity score matching, and efficacy was compared. After propensity score matching, there were no significant differences in OS and PFS between PH(−) and PH(+) patients.

There were also no significant differences in ORR and DCR by RECIST. Thus, there was no significant difference in the efficacy of Atezo + Beva with and without PH.

Incidences of AEs during treatment were compared between PH(−) and PH(+) patients before propensity score matching. The incidences of ascites, portal vein thrombus, and pyrexia were significantly higher in PH(+) patients. Ascites and portal vein thrombus can lead to decreased hepatic reserve, so diuretics, anticoagulants, and branched chain amino acids were administered to treat ascites and portal vein thrombus. This appropriate therapeutic intervention might have been the reason why there was no significant difference in OS with and without PH. In contrast, there was no significant difference in the occurrence of Grade 3–4 AEs with and without PH. Regarding varices, EIS, EVL, or B‐RTO was performed in patients with a risk of variceal rupture prior to the start of Atezo + Beva. Overall, 10% of patients presented with bleeding complications related to PH in Phase II trials of HCC using bevacizumab when preventive treatment of varices was not standardized.^36^ In the present study, rupture of varices occurred in only 2.1% of patients, but rupture of varices occurred in 1 patient who ruptured despite preventive treatment before and during Atezo + Beva and in 2 patients who were not at risk of variceal rupture before Atezo + Beva. The similarities among the three cases were their history of treatment for varices prior to HCC being noted. Patients with a history of treatment for varices should be carefully monitored, even if they do not have a risk of variceal rupture at the start of Atezo + Beva. In the present study, it is possible that the difference in variceal rupture between patients with and without PH was not significant (*p* = 0.090) because of prophylactic treatment for the risk for variceal rupture at the start of Atezo + Beva.

Limitations of this study include the retrospective design and the small sample size. In addition, EGD was performed prior to the start of Atezo + Beva in all cases, but there were several cases in which EGD for variceal evaluation was not performed again during treatment. Although these are reference findings, there were more worsening of esophagogastric varices significantly in PH(+) patients than in PH(−) patients in the follow‐up EGD (*p* < 0.001). Therefore, even if the varices had not ruptured, it is possible that cases of enlargement of varices were not assessed.

### 4.1 Conclusion

Efficacy and occurrence of Grade 3–4 AEs did not differ significantly between patients with and without PH, suggesting that Atezo + Beva can be continued in patients with PH. More to the point, prophylactic treatment of varices before the start of Atezo + Beva would allow treatment to continue relatively safely. However, it should be monitored regularly, as it might be associated with worsening of esophagogastric varices during Atezo + Beva.

## AUTHOR CONTRIBUTIONS

**Takahiro Kinami:** Conceptualization (lead); data curation (lead); formal analysis (equal); writing – original draft (equal). **shinsuke Uchikawa:** Conceptualization (equal); writing – review and editing (equal). **Tomokazu Kawaoka:** Conceptualization (equal); writing – review and editing (equal). **Shintaro Yamasaki:** Data curation (equal). **Masanari Kosaka:** Data curation (equal). **Yusuke Johira:** Data curation (equal). **Shigeki Yano:** Data curation (equal). **Kei Amioka:** Data curation (equal). **Kensuke Naruto:** Data curation (equal). **Kenji Yamaoka:** Data curation (equal). **Yasutoshi Fujii:** Data curation (equal). **Hatsue Fujino:** Data curation (equal). **Takashi Nakahara:** Data curation (equal). **Atsushi Ono:** Data curation (equal). **Eisuke Murakami:** Data curation (equal). **Wataru Okamoto:** Data curation (equal). **Masami Yamauchi:** Data curation (equal). **Daiki Miki:** Data curation (equal). **Masataka Tsuge:** Data curation (equal). **Shiro Oka:** Writing – review and editing (equal).

## FUNDING INFORMATION

There is no funding for this article.

## CONFLICT OF INTEREST STATEMENT

Authors declare no conflict of interests for this article.

## ETHICS STATEMENTS

Approval of the research protocol: The study was conducted according to the guidelines of the Declaration of Helsinki and approved by committee of epidemiological research ethics review at Hiroshima University as No. E2020‐2300 for studies involving humans. Informed Consent: Informed consent was obtained from all subjects involved in the study. Registry and the Registration No. of the study/trial: N/A. Animal Studies: N/A. Research involving recombinant DNA: N/A.

## Data Availability

The data that support the findings of this study are not publicly available due to privacy reasons.
